# Effects of halothane on the electroencephalogram of the chicken

**DOI:** 10.1002/vms3.91

**Published:** 2018-01-12

**Authors:** Amanda E. McIlhone, Ngaio J. Beausoleil, Nikki J. Kells, Craig B. Johnson, David J. Mellor

**Affiliations:** ^1^ Animal Welfare Science and Bioethics Centre/Institute of Veterinary, Animal and Biomedical Sciences Massey University Palmerston North New Zealand

**Keywords:** EEG, chicken, anaesthesia, halothane, burst suppression

## Abstract

Little is known about the effects of inhalant anaesthetics on the avian electroencephalogram (EEG). The effects of halothane on the avian EEG are of interest, as this agent has been widely used to study nociception and analgesia in mammals. The objective of this study was to characterize the effects of halothane anaesthesia on the EEG of the chicken. Twelve female Hyline Brown chickens aged 8–10 weeks were anaesthetized with halothane in oxygen. For each bird, anaesthesia was progressively increased from 1–1.5 to 2 times the Minimum Anesthetic Concentration (MAC), then progressively decreased again. At each concentration, a sample of EEG was recorded after a 10‐min stabilization period. The mean Total Power (*P*_TOT_), Median Frequency (F50) and 95% Spectral Edge Frequency (F95) were calculated at each halothane MAC, along with the Burst Suppression Ratio (BSR). Burst suppression was rare and BSR did not differ between halothane concentrations. Increasing halothane concentration from 1 to 2 MAC resulted in a decrease in F50 and increase in *P*_TOT_, while F95 increased when MAC was reduced from 1.5 to 1. The results indicate dose‐dependent spectral EEG changes consistent with deepening anaesthesia in response to increasing halothane MAC. As burst suppression was rare, even at 1.5 or 2 times MAC, halothane may be a suitable anaesthetic agent for use in future studies exploring EEG activity in anaesthetized birds.

## Introduction

Anaesthetics are used to induce unconsciousness and loss of muscle tone during surgical or experimental procedures (Antognini & Carstens 2002). In avian clinical practice inhalant anaesthetics are preferred over injectable agents for their rapid induction and recovery, facilitating easy and safe adjustment of anaesthetic concentration (Ludders & Matthews 2007; Lierz & Korbel 2012). While the effects of different inhalant anaesthetics on the electroencephalogram (EEG) have been extensively investigated in mammals (e.g. Johnson & Taylor 1998; Antunes *et al*. 2003; Orth *et al*. 2006; Murrell *et al*. 2008), less is known about the effects of different anaesthetics on the avian EEG. The effects of halothane on the avian EEG are of particular interest, as this agent has been widely used to study nociception and analgesia in mammals (Murrell & Johnson 2006). The development of a similar model to study pain in avian species would be beneficial from an animal welfare perspective.

Electrical activity in the brain, particularly the cerebral cortex, can be monitored using an EEG (Murrell & Johnson 2006). In mammals, increasing depth of anaesthesia is generally associated with EEG synchronization, characterized by a shift towards high amplitude, low frequency activity (Clark & Rosner 1973; Otto 2008). Such EEG changes may be quantified, for example, by calculating variables such as the median frequency (F50), spectral edge frequency (F95) and total power (Murrell *et al*. 2008). Furthermore, at high concentrations some anaesthetic agents cause burst suppression (BS), identified as periods of isoelectric EEG interspersed with high‐voltage bursts of activity (Steriade *et al*. 1994; Yoshitani *et al*. 2003; Otto 2008).

The minimal alveolar concentration (MAC) is defined as the minimum concentration of an anaesthetic required to prevent purposeful movement in response to a supramaximal noxious stimulus in 50% of subjects and is an index of anaesthetic potency (Quasha *et al*. 1980). In birds, where gas exchange occurs in parabronchi instead of alveoli, MAC is instead referred to as minimum anaesthetic concentration (Ludders *et al*. 1989). In both cases, MAC is usually measured in terms of volume %. Volatile anaesthetics exert their effects through independent actions at both the brain and spinal cord levels (Antognini 1997). Evidence indicates that MAC relates to direct action at the spinal level to suppress movement reflexes (Antognini & Schwartz 1993; Rampil *et al*. 1993; Antognini 1997), therefore not necessarily reflecting effects on brain activity, as assessed using the EEG.

In mammals, different anaesthetic agents are known to exert different effects on the EEG at equivalent levels of MAC. For example, halothane causes less EEG suppression in man, rats, horses and cats than equipotent MAC multiples of isoflurane, sevoflurane, methoxyflurane or desflurane (Thomsen & Prior 1996; Johnson & Taylor 1998; Tsushima *et al*. 1998; Orth *et al*. 2006; Murrell *et al*. 2008). In addition, the EEG effects of increasing MAC multiples have been shown to vary between agents and species (Johnson & Taylor 1998; Antunes *et al*. 2003; Murrell *et al*. 2008).

The mammalian and avian EEG are of similar appearance and share some unique characteristics; for example, birds and mammals are the only animals that exhibit slow wave sleep (SWS) or rapid eye movement (REM) sleep waveforms (Rattenborg 2006; Lesku *et al*. 2011). However, the effects of volatile anaesthetics on the EEG may differ, due to differences in both respiratory (Ludders 2015) and neural (Butler & Cotterill 2006; Rattenborg 2006) anatomy and physiology. The quantitative effects of different isoflurane, sevoflurane and methoxyflurane concentrations on the chicken EEG have been investigated in two previous studies (Martin‐Jurado *et al*. 2008; McIlhone *et al*. 2014). However, to the best of the authors’ knowledge, no previous studies have examined the effects of halothane on the chicken EEG. The objective of this study was to quantify the effects of three different concentrations of halothane MAC on the chicken EEG.

## Materials and methods

Twelve 8‐ to 10‐week‐old Hyline Brown female chickens were used. The chickens were sourced from a commercial caged layer farm a minimum of 2 days prior to the start of the experiment. Chickens were housed indoors in groups of 5–7 on wood shaving substrate under controlled temperature and light conditions (20°C, 12‐h light/dark cycle). Chick Starter Crumbles (Inghams Enterprise NZ, Levin, New Zealand) and water were available *ad libitum*. At the conclusion of the study, all chickens were re‐homed to free‐range lifestyle blocks. All procedures were carried out in accordance with the Massey University Code of Ethical Conduct for the Use of Live Animals in Research, Testing and Teaching, and with the approval of the Massey University Animal Ethics Committee, protocol number 09/09.

### Anaesthesia

Halothane (Nicholas Piramal India Ltd, Maharashtra, India), vaporized in oxygen, was administered to effect using either a chamber or a face mask. Once anaesthesia was judged adequate (loss of righting reflex and muscle tone), intubation was carried out. To aid intubation, lidocaine (0.1–0.2 mL, Nopaine; Phoenix Farm Distributors Ltd, New Zealand) was applied to the laryngeal opening, and a 2.5 mm non‐cuffed endotracheal tube was inserted into the trachea. Halothane anaesthesia was maintained using a t‐piece non‐rebreathing anaesthetic circuit (Mapleson E) and intermittent positive pressure ventilation using a mechanical thumb ventilator (V‐valve ventilator, Vetronics, Bioanalytical Systems Inc., IN, USA).

Inspired and end‐expiratory halothane and carbon dioxide (CO_2_) concentrations (volume %), and respiration rate were monitored and recorded throughout using an anaesthetic monitor (Hewlett‐Packard M1025B; Hewlett‐Packard, Germany), which was calibrated daily according to the manufacturer's instructions. Gas was sampled at a rate of 90 mL min^−1^ from the system end of the endotracheal tube, using a low dead‐space connector. Ventilatory responses were adjusted as required to maintain end‐tidal CO_2_ within the normal physiological range. The chicken was placed on a water‐heated mat maintained at 37°C and covered with a polypropylene blanket to reduce heat loss. Heart rate was monitored via ECG recording (see below) and body temperature was monitored using a cloacal thermocouple.

Halothane was delivered at three end‐tidal concentrations (volume %) based on 1, 1.5 and 2 times MAC. The MAC value of 0.8% used in this study was derived from Ludders *et al*. (1988). The multiples of 1, 1.5 and 2 MAC were, therefore 0.8, 1.2 and 1.6% (all ±0.1 vol%), respectively. Halothane concentration was sequentially increased, then decreased (i.e. 1 MAC ascending, followed by 1.5 MAC ascending, 2 MAC, 1.5 MAC descending and 1 MAC descending). At each halothane concentration, anaesthesia was held stable for 10 min (i.e. anaesthetic concentration did not fluctuate outside the 0.05% sampling range for more than 10 s), before 15 min of EEG were recorded for analysis. At the end of each sampling period, the vaporizer was adjusted to the next halothane concentration.

### EEG and ECG recording

The EEG was recorded using four 27‐gauge subcutaneous, stainless‐steel needle electrodes (Viasys Healthcare, UK). Following induction of anaesthesia, electrodes were positioned to record the EEG from the left and right sides of the skull using two separate channels on the chart recorder. The electrode montage used was based on that described for horses (Mayhew & Washbourne 1990). The electrode sites on the chickens were: lateral to the comb (non‐inverting electrodes) and caudal to the external auditory meatus (inverting electrodes). The ECG was recorded on a separate channel, using a base‐apex configuration with electrodes located medio‐cranial to the keel bone and caudal to the sternum, and a common earth electrode (EEG and ECG recording) located lateral to the pelvis.

The electrode cables were fed via individual break‐out boxes (one for each channel) to separate amplifiers (Iso‐Dam isolated physiological signal amplifier, World Precision Instruments, Sarasota FL, USA). The signals were amplified with a gain of 1000 and band‐pass filtered between 0.1 Hz and 0.1 kHz. Each amplifier fed into an analogue‐to‐digital converter (Powerlab; ADInstruments Ltd, Australia) which digitized the signals at 1000 points/second and displayed and stored them on an Apple personal computer using LabChart 5.5.6 recording software (ADInstruments Ltd, Australia).

### Analysis of the EEG

EEG data were analysed to determine the burst suppression ratio (BSR) and were also transformed for frequency analysis. The BSR is the ratio of time spent in isoelectric EEG to time spent in active EEG and is expressed as a percentage. Isoelectric EEG was defined as EEG with an amplitude 1/8 or less of the active EEG amplitude (Gibson *et al*. 2009). BSR was calculated over a 120‐s period in the middle of the EEG recording by dividing the number of seconds of burst suppression by 120 and multiplying by 100. A period of burst suppression was defined as a segment of isoelectric EEG equal to or greater than 0.5 s in duration. Similarly, to be classified as active EEG or burst activity, the EEG waveform needed to persist for 0.5 s or more. Single or small groups of spike activity were occasionally present during isoelectric periods (burst suppression). These were only considered to be active EEG if they lasted for 0.5 s or more.

The EEG was also subjected to Fast Fourier Transformation (FFT) using purpose‐written software (Spectral Analyser, CB Johnson, Massey University, New Zealand), which calculated the frequency spectrum for consecutive non‐overlapping 1 s epochs. The frequency spectrum is a graphical representation of the contribution that each frequency makes to the power of the EEG waveform. Analysis of the EEG is based on the area under the frequency spectrum graph. Variables derived from the frequency spectrum were: the total power (*P*_TOT_), which is the total area under the frequency spectrum; the median frequency (F50), which is the frequency below which half the total power is located; and the 95% spectral edge frequency (F95), which is the frequency below which 95% of the total power is located (Murrell & Johnson 2006).

Five minutes of EEG, from the middle of each recording period, were used for frequency analysis. Segments containing burst suppression were excluded from this analysis. The mean *P*_TOT_, F50 and F95 were calculated for each sample period and subjected to statistical comparison.

### Statistical analysis

All data were tested for normality (skewness and kurtosis of residuals distribution and Shapiro–Wilk tests) to ensure they met the assumptions for parametric analysis. Data were analysed using one‐way ANOVA with repeated measures for anaesthetic concentration. Where a significant effect of concentration was found, post hoc pairwise comparisons were performed with Bonferroni adjustment for multiple comparisons. Mean *P*_TOT_ data were not normally distributed, therefore were log transformed prior to analysis. All analyses were conducted using SAS version 9.2 (SAS Institute Inc., NC). Differences were considered significant at *P *<* *0.05. Data are presented as least square means ± standard deviation, unless otherwise stated.

## Results

The results from both channels (left and right cortices) were equivalent in both burst suppression ratios and EEG frequency analysis. The results from channel one (left side of the brain) only are reported.

### Burst suppression

Burst suppression was observed in 4/12 chickens at 2 MAC (BSR 0.87, SD 1.84%) and in one chicken during 1.5 MAC descending (BSR 0.03, SD 0.12%). No burst suppression occurred at 1 MAC (ascending or descending) or 1.5 MAC ascending. Despite an overall effect of concentration [*F*(4,44) = 2.66; *P *=* *0.045], there were no significant differences in BSR between halothane concentrations (adjusted *P *≥* *0.13).

### EEG frequency analysis

**Figure 1:**
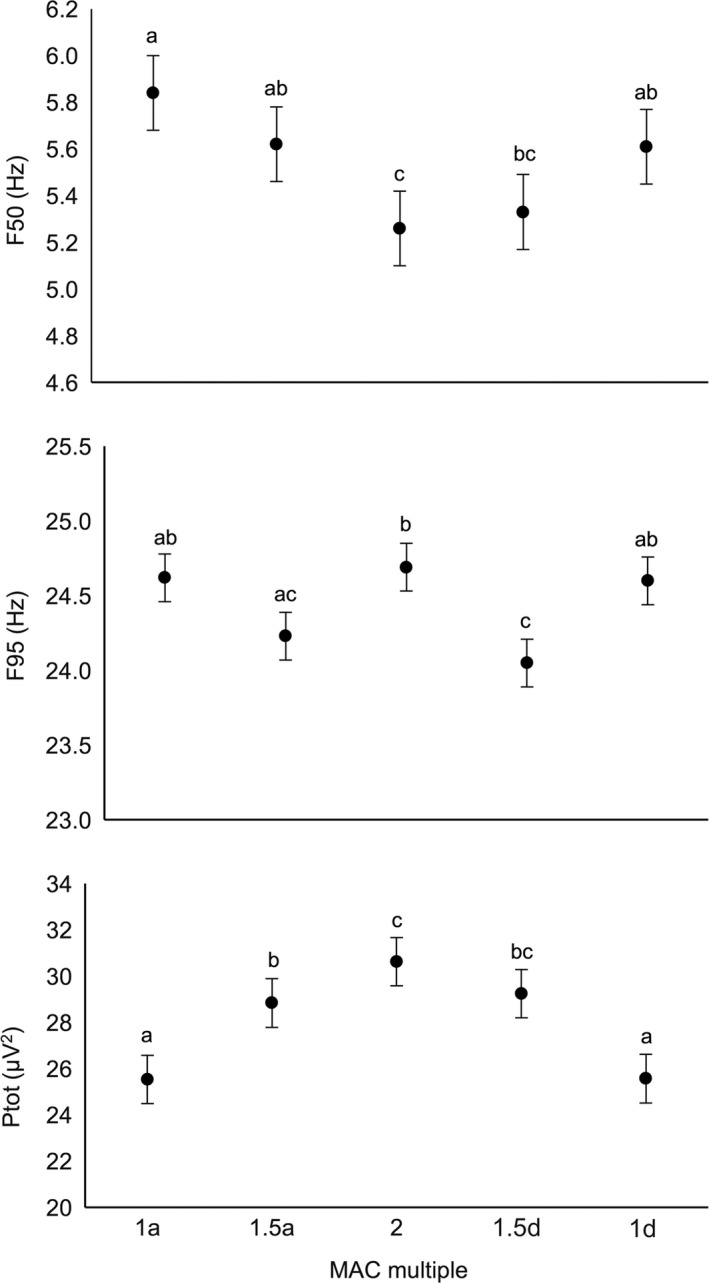
Comparison of mean (±standard error) a) median frequency (F50), b) 95% spectral edge frequency (F95) and c) total power (*P*_TOT_) of the chicken EEG at different ascending (a) or descending (d) halothane minimum anaesthetic concentration (MAC) multiples. Within each graph, values with different superscript letters are significantly different (*P *<* *0.05).

Halothane concentration had a significant effect on mean F50 [*F*(4,44) = 8.48; *P *<* *0.0001], F95 [*F*(4,44) = 7.62; *P *<* *0.0001] and *P*_TOT_ [*F*(4,44) = 48.36; *P *<* *0.0001]. Mean F50 tended to decrease with increasing halothane concentration and was significantly lower at 2 MAC than 1 MAC (descending or ascending) or 1.5 MAC (descending only; Fig. 1a). The relationship between halothane concentration and mean F95 was non‐linear; F95 at 1.5 MAC (descending and ascending) was lower than 2 MAC, whereas F95 did not differ between 1 MAC (descending or ascending) and 2 MAC (Fig. 1b). Mean *P*_TOT_ (back‐transformed) tended to increase with increasing halothane concentration and was significantly higher at 2 MAC than 1 MAC (descending or ascending) or 1.5 MAC (descending only; Fig. 1c).

## Discussion

To the best of our knowledge, this study is the first to quantify the effects of different MAC multiples of halothane on the avian EEG. In general, increasing halothane concentration resulted in a dose‐dependent decrease in F50 and increase in *P*_TOT_. Burst suppression was not observed until halothane concentration was increased to 2 MAC, when it was observed in four birds.

The effects of halothane on the chicken EEG are broadly consistent with those reported for mammals. For example, dose‐dependent reductions in F50 and F95 with increasing halothane concentration were reported in humans (Thomsen & Prior 1996), rats (Antunes *et al*. 2003) and horses (Johnson *et al*. 1994; Johnson & Taylor 1998) across the concentration range of 0.6–1.5 MAC. In contrast, Murrell *et al*. (2008) reported a variable increase in F50 and no change in F95 in rats anaesthetized with halothane over the range 1.25–1.75 MAC. This study differed, however, in that electrical recordings were made from the cortical surface using surgically implanted electrodes located over the left and right primary somatosensory (S1) cortices. It is possible that brain activity contributing to the decrease in F50 and F95 reported in other studies arises, at least in part, from cortical areas other than S1. While *P*_TOT_ has not been reported by studies investigating the EEG effects of halothane, the increase in *P*_TOT_ observed with increasing halothane concentration in chickens is consistent with that reported in humans anaesthetized with isoflurane, desflurane or sevoflurane (Schwender *et al*. 1998).

Unlike some other reports, the changes in F95 observed in chickens in this study did not follow a linear dose‐response pattern. While the initial reduction in F95 between 1 and 1.5 (descending) MAC, and subsequent increase in F95 between 1.5 (descending) and 1 MAC, was typical of that expected with increasing/decreasing anaesthesia, the significant increase in F95 between 1.5 and 2 MAC was unexpected. However, previous studies in mammals have not examined quantitative EEG responses at 2 MAC halothane; therefore, a similar response pattern in the mammalian EEG cannot be ruled out.

The occurrence of burst suppression in this study contrasts with EEG studies in humans, rats and horses in which no burst suppression was reported over the range 0.6–1.75 MAC halothane (Johnson *et al*. 1994; Thomsen & Prior 1996; Johnson & Taylor 1998; Antunes *et al*. 2003; Murrell *et al*. 2008). However, in this study, burst suppression was not evident until halothane concentration was increased to 2 MAC, and occurred in one‐third of chickens at this concentration. Only one other study has examined the cerebral effects of 2 MAC halothane; Tsushima *et al*. (1998) reported no burst suppression in the cat EEG at either 1.3 or 2 MAC. Given the lack of additional data on mammalian responses to 2 MAC halothane, it is difficult to draw conclusions regarding similarities or differences in avian and mammalian EEG responses to deep halothane anaesthesia. It is therefore possible that the burst suppression observed at this concentration in our study may have been a function of agent concentration, rather than a differential effect of halothane on the avian brain. Further study is required to confirm this.

Based on reported BSRs at equipotent concentrations, halothane appears to cause less suppression of the chicken EEG than either isoflurane or sevoflurane (Martin‐Jurado *et al*. 2008; McIlhone *et al*. 2014), although a direct comparison would be required to confirm this. The results of this study, along with previously reported EEG responses to isoflurane, sevoflurane and methoxyflurane in the chicken (Martin‐Jurado *et al*. 2008; McIlhone *et al*. 2014), suggest that inhalant anaesthetics have similar actions in both the avian and mammalian brains.

The EEG can also be used as a tool to study noxious stimulation. Noxious stimuli elicit changes in the activity of the cerebral cortex under light general anaesthesia, with these changes being attenuated or abolished with deepening anaesthesia (Otto 2008). In mammals, the minimal anaesthesia model (Murrell & Johnson 2006) has been used extensively to study the EEG effects of noxious stimuli and to compare the efficacy of different analgesic regimens (e.g. Johnson *et al*. 1999, 2005; Murrell *et al*. 2003; Gibson *et al*. 2007; Kongara *et al*. 2014). Halothane is the maintenance agent of choice in this model, as it is not considered to have anti‐nociceptive properties (England & Jones 1992), and causes less suppression of brain activity at concentrations required to achieve a surgical plane of anaesthesia than isoflurane, sevoflurane, desflurane or methoxyflurane (Johnson & Taylor 1998; Tsushima *et al*. 1998; Antunes *et al*. 2003; Murrell *et al*. 2008). In chickens, it appears that halothane and methoxyflurane (McIlhone *et al*. 2014) produce less burst suppression of the EEG at equipotent concentrations than isoflurane or sevoflurane. However, methoxyflurane has known analgesic properties (Lambie 1963; Coffey *et al*. 2014), thus limiting its use in studies of nociception or analgesia. Therefore, halothane may be the most appropriate agent to use in future experimental studies of brain responses to external stimuli in birds.

## Conclusions and clinical relevance

The avian brain appears to respond in a similar manner to halothane anaesthesia as the mammalian brain, with dose‐dependent changes in the EEG occurring in response to changing halothane concentration. In chickens, burst suppression was absent at 1 or 1.5 times MAC, indicating little suppression of brain activity at these concentrations. Halothane may therefore be an appropriate agent for future experimental studies of avian brain responses to external stimuli.

## Source of Funding

This research was not funded by a specific project grant.

## Conflicts of interest

The authors declare no potential conflict of interest.

## Ethical statement

The authors confirm that the ethical policies of the journal, as noted on the journal's author guidelines page, have been adhered to and the appropriate ethical review committee approval has been received. The Massey University Code of Ethical Conduct for the Use of Live Animals in Research, Testing and Teaching was followed.

## Contributions

Study design: AM, NB, CJ, DM. Data collection and analysis: AM, CJ. Statistical analysis: AM, NB, NK. Draft manuscript preparation: NB, NK, CJ, DM. Manuscript revision and approval: AM, NB, NK, CJ, DM.
